# Thermal variation in human temporal bone using rigid endoscope

**DOI:** 10.1016/j.bjorl.2023.101381

**Published:** 2023-12-19

**Authors:** Thales Xavit Souza e Silva, Aline Bruno Figueiredo Nicolau, Marcos Luiz Antunes

**Affiliations:** Universidade Federal de São Paulo, Departamento de Otorrinolaringologia e Cirurgia de Cabeça e Pescoço, São Paulo, SP, Brazil

**Keywords:** Endoscope, Temporal bone, Thermal variation

## Abstract

**Objective:**

Compare thermal variation in the region of the External Acoustic Canal (EAC) and the Round Window (RW) using different rigid endoscopes and light sources in human temporal bones.

**Method:**

This is an analytical experimental study using human temporal bones. Thermal variation was assessed during ten minutes, using a thermometer in the region of the EAC and the RW of two temporal bones, right and left. We used three different endoscopes (0° 4-mm, 0° 3-mm and 30° 4-mm) and five intensity/type light source (Halogen 100%, LED 50%, LED 100%, Xenon 50% and Xenon 100% with the same endoscope light fiber.

**Results:**

We found temperature elevations in the EAC and RW in all measurements. Larger caliber endoscopes (4-mm) and light sources at 100% intensity generated higher temperatures, slightly higher in halogen and xenon. The 30° endoscopes tended to put more heat on structures, with little difference in most measurements. We identified greater temperature variations in the EAC of the right and left temporal bones compared to the RW overall. The highest temperature acquired in the present study was in the EAC of the temporal bone with a 4-mm and 30° endoscope, using xenon light source (intensity of 100%), with an increase of 4.51 °C.

**Conclusion:**

The type of endoscope and light source can influence the thermal variation and the risk of tissue injury during endoscopic ear surgery. Larger endoscopes with xenon and halogen light sources at maximum intensity generate more heat.

**Level of evidence:**

5.

## Highlights

• Endoscopes can generate thermal injury and tissue functional impairment.
• Hyperthermia can influence the responses both cochlear nerve and the brainstem.
• Caliber and angulation of the endoscope influences the thermal variation.
• Type and intensity of the light source influences the thermal variation.
• Large-caliber endoscopes with xenon or halogen light generate more heat.

## Introduction

Endoscopic ear surgeries have expanded in recent years, both for diagnosis and for surgical treatment of ear diseases. The use of surgical endoscopes allows good illumination and enlargement of the surgical field, which allows to approach the anatomical sites that are less accessible through the use of direct view or surgical microscope, offering a “wide eyepiece” type of view, especially by having a peripheral view with angled endoscopes and by having less necessity of manipulating neighboring structures in order to reach the surgical field.^1^, ^2^, ^3^, ^4^

One of the concerns about the use of endoscopes in the ears is the theoretical risk of thermal injury and tissue functional impairment caused by light source coupled with the optics, even though there are few studies that quantify or pinpoint the sites of temperature elevation in the ear.^1^, ^2^

A few research have demonstrated that cochlear function can be modified by body or cochlear temperature; in other words, hyperthermia can influence the responses both cochlear nerve and the brainstem, and one of the possible alterations is the decrease in interpeak interval of the Brainstem Auditory Evoked Potential (BAEP).^5^

The rigid endoscopes that are often used for ear surgery have a diameter of 2.7 mm, 3 mm and 4 mm, besides the angle of 0°, 30°, 45° and even 70°. The larger the diameter, the better the image quality, but the smaller the space for manipulating the surgical field. When it comes to light sources, the xenon, halogen, and Light Emitting Diode (LED) are the ones commonly used.^2^

What needs to be better understood is whether the use of endoscopes in ear surgeries, with the particularities of the diameter, the angle of the optics and the type and intensity of light source, directly influences tissue temperature and the risks for the structural functions. Background: The aim of the study was assessing thermal variation concerning most used rigid endoscopes and light sources in outpatient practice and in endoscopic middle ear surgery, to avoid thermal injuries to human temporal bones structures.

## Methods

**Figure 1:**
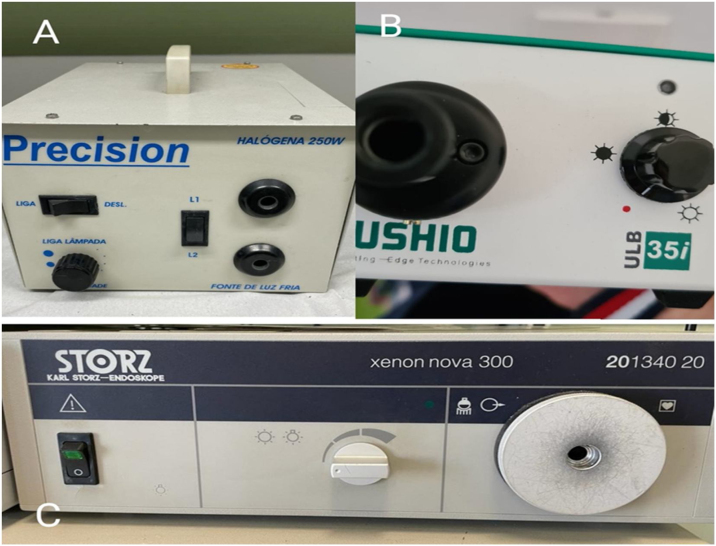
Light sources used. (A) Halogen. (B) Light Emitting Diode (LED). (C) Xenon.

This is an analytical experimental study using human temporal bones. We used rigid endoscopes of 3 mm and 0°, 3 mm and 30°, 4 mm and 0°, 4 mm and 30°, with three types of light sources in different intensities, namely: halogen (Precision®, 250 W) at 100%, LED (USHIO®, ULB-35) at 100% and 50%, xenon (Storz® – Xenon nova 300) at 100% and 50%. For all light sources, the same standard light cable was used (Storz® 3.5 mm-cristal point). We did not use a 50% halogen light source because this light is increasingly falling in disuse in surgeries, mainly because of its weaker intensity and color (Fig. 1).

**Figure 2:**
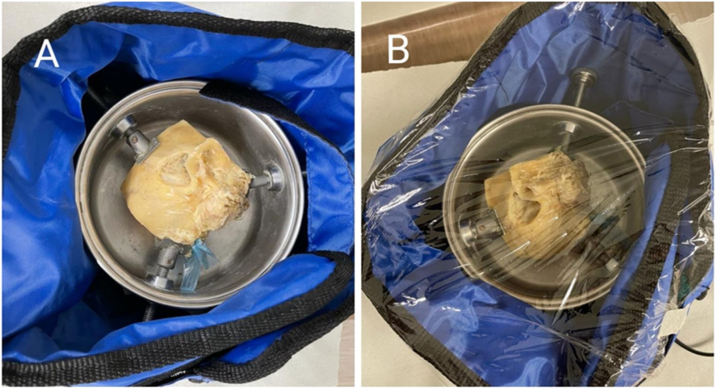
Right temporal bone wrapped in thermal blanket. (A) Without plastic PVC film. (B) With PVC plastic film.

We selected two temporal bones, a right one and a left one, which belong to the temporal bone dissection laboratory of the Otorhinolaryngology Service of the Federal University of São Paulo (UNIFESP); in order to try to reproduce the temperature of the human body and to achieve more reliable situations in relation to the body in *vivo*, we wrapped the temporal bones with a thermal blanket (OPTEK – TERMOTEK), covered them with plastic PVC film and fixed them with adhesive tape, and started measuring the temperatures after the temporal bone reaches and maintains stability at a temperature of approximately 36 °C (Fig. 2).

**Figure 3:**
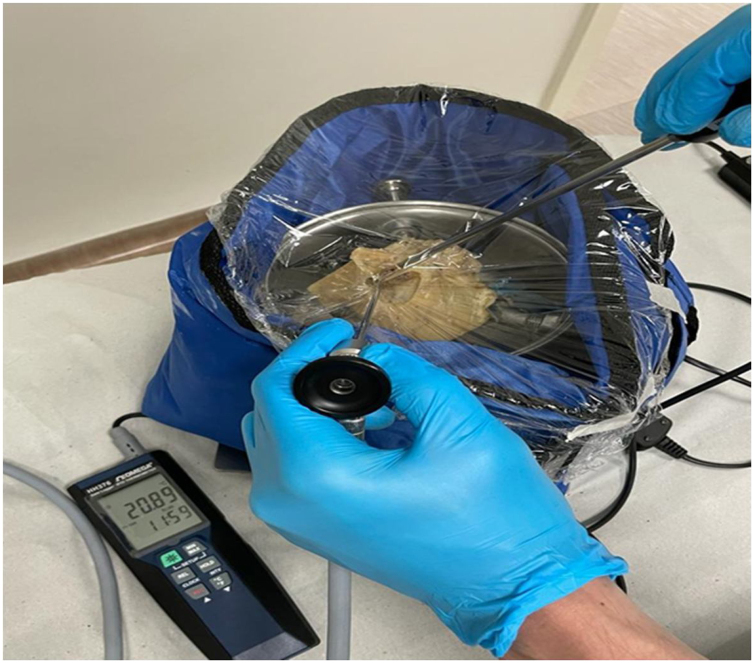
Endoscope and thermometer inserted into the temporal bone through the perforations made in the PVC plastic film.

We made markings with a permanent marker brush of the places where the endoscope and probe would be applied in the External Acoustic Canal (EAC) and Round Window (RW) to avoid loss of the analyzed anatomical site. We made perforations in the PVC film and introduced 0.5 cm of the endoscopes into the bone portion of the EAC, without touching the bone wall. For measurements in the RW, we introduced 1.0 cm of the endoscopes into the EAC, next to the tympanic annulus (Fig. 3).

**Figure 4:**
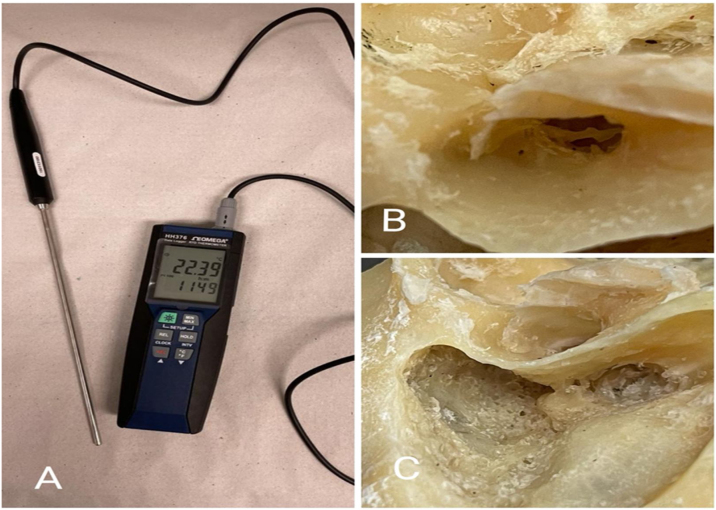
Thermometer used and preparation of the temporal bone. (A) Omega® HH376 thermometer with 5.0 mm diameter probe. (B) External Acoustic Canal (EAC) showing absence of tympanic membrane, with preserved ossicular chain. (C) Posterior tympanotomy region (red arrow).

A thermometer (Omega® HH376 with a 5.0 mm diameter probe) was used to register the temperature variation with an accuracy of 0.01 °C in the two different anatomical regions of the temporal bones. For EAC analysis, the thermometer sensor was parallel to the optics, and we removed the tympanic membrane, previously preserving the ossicular chain. For the RW, we made a posterior tympanotomy since there was not enough space and angulation for the optic and probe together. Subsequently, we introduced the sensor through the latter, leaving it placed and touching the upper portion of the RW. We took temperature notes every minute for all types of endoscopes and light sources used, starting at zero time (T0), and going up to ten minutes time (T10) (Fig. 4).

Were made a total of eighty combinations of situations with different endoscopes (angulation and caliber), types and intensities of light sources, laterality of the temporal bone and anatomical region (EAC and RW).

Statistical analysis: Statistical and graphic analysis were performed on a computer using specific software (Excel-Microsoft 365®). The thermal variation was only described in number of Celsius degrees in each measurement between T0 and T10 times.

## Results

**Figure 5:**
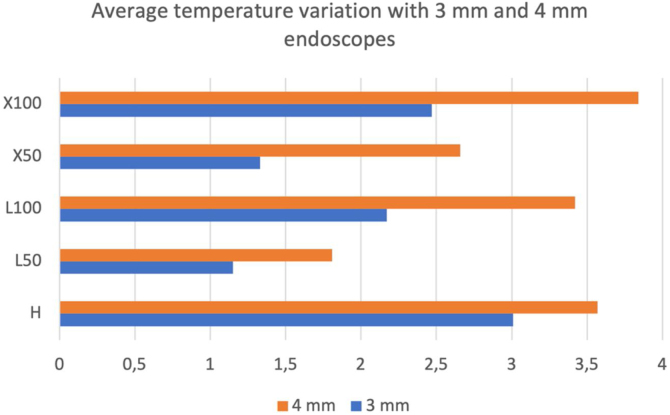
Average temperature variation with 3-mm and 4-mm endoscopes. H: 100% halogen light. L50: LED at 50%. L100: LED at 100%. × 50: xenon at 50%. × 100: 100% xenon.

We obtained temperature elevations in the EAC and RW in all measurements after approaching the endoscopes, especially when using the 4 mm in comparison to the 3 mm ones (Fig. 5). In addition, light sources with 100% intensity generated higher temperatures, slightly higher in halogen and xenon, with no apparent considerable predominance of one over the other.

The maximum temperature average was using 4-mm optic with xenon light source in 100% intensity (Fig. 5).

**Figure 6:**
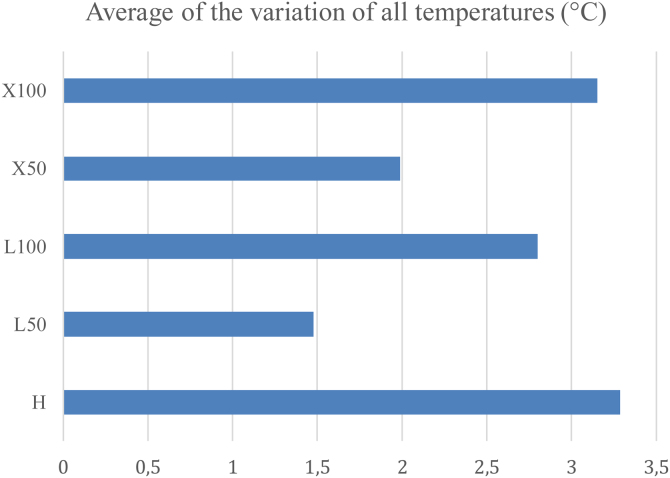
Average of the variation of all temperatures. H: 100% halogen light. L50: LED at 50%. L100: LED at 100%. × 50: xenon at 50%. × 100: 100% xenon.

The Fig. 6 represents the average of all temperatures using different light sources and intensities independent of diameter of the rigid endoscope. This is the global average temperature. Note that the average was very similar between xenon and halogen light source at 100%.

In general, xenon sources produced more heat when compared to the LED, with ten measurements showing a difference greater than 1 °C, five of which were at 50% and the others were at 100% of maximum light intensity. There was no 100% xenon predominance in relation to halogen, as their average temperature variation was very similar; besides, higher temperatures occurred in eight occasions in xenon and also in eight occasions in halogen. Despite the seeming similarity in this last result, the same does not occur when halogen light is compared to LED at 100%. The former produced more heat in the anatomical structures studied and, in five measurements, this value reached more than 1 °C of difference.

As for the laterality of the temporal bones studied, we did not observe significant differences between the right and left sides. In only five measurements the difference was greater than 1 °C, in three of them greater on the left and in two, greater on the right. In the other measurements, each time one side was predominant, with a difference of less than 1 °C. And in most cases, this difference was insignificant, not even 0.1 °C of difference.

We used Graphs 3 and 4 to exemplify the comparisons between 0° and 30° endoscopes of the same caliber. The graphs correspond to the temperature curve in the RW of the left temporal bone. We concluded that those at 30° tended to heat up the structures more, with little difference in most measurements. However, in eight measurements out of the eighty in total, this difference was greater than 1 °C.

**Figure 7:**
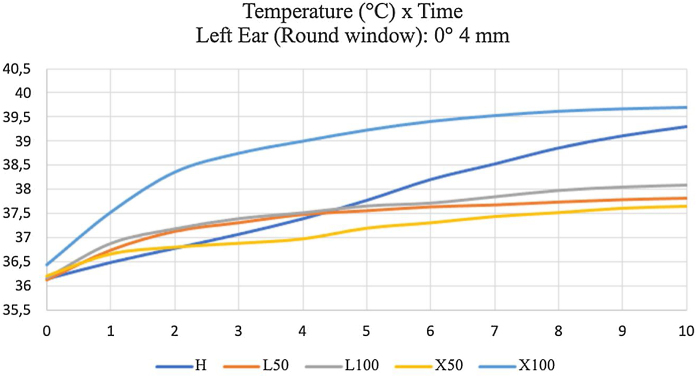
Temperatures measured for ten minutes in the round window of the left temporal bone, using a 0°- and 4-mm endoscope. H: 100% halogen light. L50: LED at 50%. L100: LED at 100%. × 50: xenon at 50%. × 100: 100% xenon.

It shows that that the most thermal variation was with 100% halogen light and 100% xenon, and the increase was faster with the halogen. The different temperature between LED at 50% and xenon at 50% was low, and LED at 100% was lower than xenon at 100% (Fig. 7) with 4 mm, 0° rigid endoscope.

**Figure 8:**
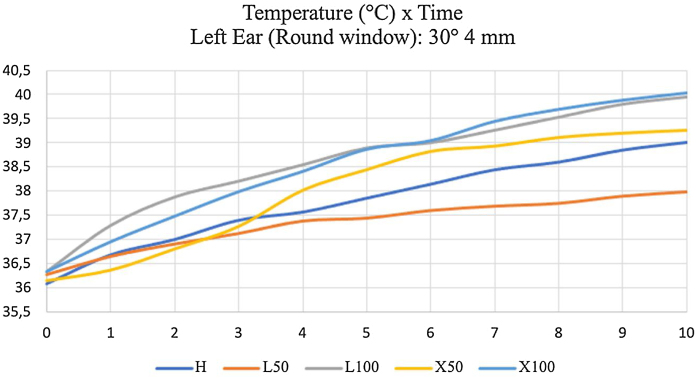
Temperatures measured for ten minutes in the round window of the left temporal bone, using a 30°- and 4-mm endoscope. H: 100% halogen light. L50: LED at 50%. L100: LED at 100%. × 50: xenon at 50%. × 100: 100% xenon.

It shows that final temperature (T10) was similar between Halogen and LED at 100% (about 40 °C). The lowest temperature was obtained with LED at 50% with 30° 4 mm rigid endoscope (Fig. 8).

**Figure 9:**
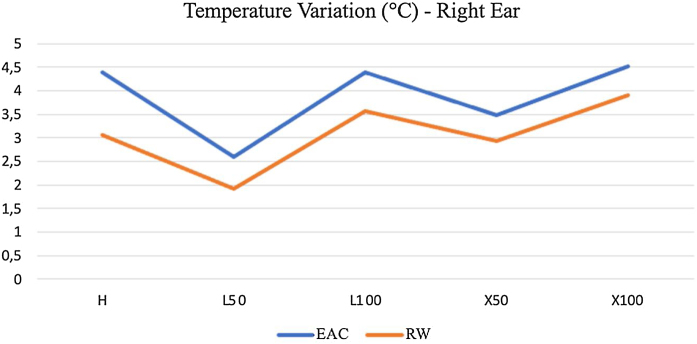
Temperature variation in the right temporal bone with a 30° and 4-mm endoscope. H: 100% halogen light. L50: LED at 50%. L100: LED at 100%. × 50: xenon at 50%. × 100: 100% xenon. EAC, External Acoustic Canal; RE: Right temporal bone.

When it comes to the anatomical location studied, we identified greater temperature variations in the EAC of the right and left temporal bones compared to the RW in general. Fig. 9 exemplifies this finding, representing the right temporal bone, when using the endoscope that generally produced more heat, that is, the 4 mm and 30° endoscope. In fact, the highest temperature verified in the present study was in the EAC of the temporal bone on this side with a 4 mm and 30° endoscope and 100% xenon light source, with an increase of 4.51 °C, reaching 40.73 °C at the end of ten minutes.

**Figure 10:**
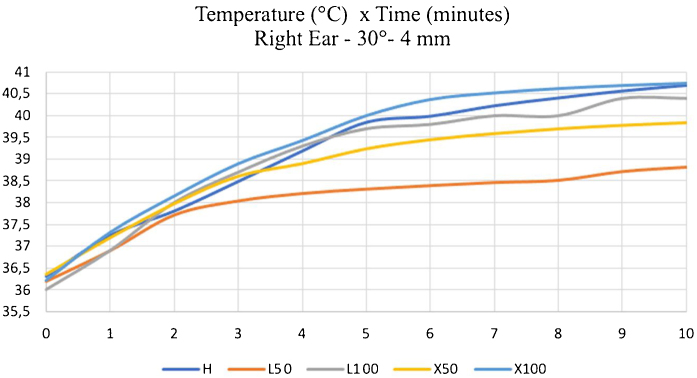
Temperatures measured for ten minutes in the external acoustic meatus of the right temporal bone, using a 30° and 4-mm endoscope. H: 100% halogen light. L50: LED at 50%. L100: LED at 100%. × 50: xenon at 50%. × 100: 100% xenon. EAC, External Acoustic Canal; RE, Right Ear.

Another point is the speed at which temperatures reached the peak tendency. At first, temperatures rose abruptly and, as the minutes passed, the speed reduced, and the temperature could reach stability before ten minutes of analysis in some measurements. This finding is more clearly, as verified when studying the EAC temperature of the right temporal bone with a 4 mm and 30° endoscope (Fig. 10). In this case, we noticed a tendency towards stability six minutes after turning on the light source.

## Discussion

Our study aimed at combining types and intensities of light sources, types of endoscopes and ear laterality to simulate different situations of heat generation that may occur during the endoscopic surgery of the ear. Our aim was to assess under which conditions ear surgeries can be performed more safely, effectively and with less tissue impairments.

There are several studies in the literature that evaluate the effects of heat generated by endoscopes in otorhinolaryngology, especially with emphasis on sinonasal structures, with few studies on ear structures. However, a study showed that when an endoscope with a xenon light source was introduced into the middle ear for a specific period, the inner ear function deteriorated, as confirmed by audiological tests.^6^

In the present study, we noticed an increase in temperature in the EAC and in the RW of the human temporal bones when we introduced all types of tested endoscopes, especially the larger ones, increasing the risk of tissue damage and unwanted symptoms, such as vertigo, since the elevation of temperature can often be enough to produce even caloric stimulation in the lateral channels.^7^

We registered the highest thermal variation with a 4 mm, 30° endoscope, with a 100% xenon light source, with an elevation of 4.51 °C after 10 min of analysis. Most studies show considerably higher values, as they use as a method of analysis the thermal variation at the endoscope tip, and not in the tissues manipulated during surgery. It explains the different increase of temperature between our study and previous studies. And despite the lower temperature variation verified in the present study, the risk of tissue injury remains.^8^

The highest temperatures at the tip of the endoscopes were achieved with those one most caliber, reaching a temperature of the order of 104.6 °C in 4 mm endoscopes. Therefore, greater care is needed when approaching anatomical structures, especially avoiding direct contact of the endoscope tip with the soft tissues.^3^, ^6^, ^7^

As for the type and intensity of light, we used halogen, LED and 100% xenon sources, besides LED and 50% xenon sources. We obtained higher temperatures in the sources at maximum intensity, mainly in the halogen and xenon sources. Tomazic et al. studied the thermal effects produced by 4 mm endoscopes (0° and 30°) with halogen, LED and xenon light sources in the nasal cavity and found similar results.^9^

Other studies also describe higher temperatures with light at 100% intensity when evaluating LED and xenon light, suggesting that for greater safety in the surgical procedure, the ideal would be to use the lowest light intensity possible in the field without compromising good visibility.^2^, ^10^

In the present study, halogen light generated temperature elevations like those of xenon and higher than those of LED at maximum intensity. Hensman et al. studied the thermal variation in endoscopes and light cables using halogen and xenon light sources and reported that there was no significant difference in the temperature generated by these two sources at the end of the analysis.^11^ However, other works observed lower temperatures in the halogen light sources. Even being cooler, they did not provide satisfactory light for high-definition cameras during nasal endoscopic surgeries; therefore, they would not be ideal for the surgical procedure. We must consider, that many services use halogen light sources in outpatient clinics because they are of lower cost, and with maximum intensity to allow better vision, which can lead to a potentially harmful temperature increase in these situations.^6^, ^9^

Regarding laterality, we observed no significant difference in the thermal variation between right and left ears and, therefore, the side does not seem to be a risk factor to be considered to avoid tissue injury. We did not find studies in the literature comparing temperatures between the sides of the ears to corroborate our finding, but we believe this type of analysis is important.

We questioned whether the angulation of the endoscope tip could exert any influence on the generation of heat in the manipulated tissue. Then, we verified the temperatures reached with the 0° and 30° endoscopes, the most used in the practice of endoscopic ear surgery. When comparing endoscopes of the same caliber with each other, we observed temperatures slightly higher in those of 30° of angulation, except in eight measurements, in which the temperature difference was greater than 1 °C. Other studies corroborate our findings, in which they describe higher temperatures in endoscopes of 30°, when compared to those of 0°, with a slight difference (on the order of 0.4 °C–1.2 °C) in the minimum temperatures verified and more evident (on the order of 4 °C–5.8 °C) at maximum temperatures in all three types of light evaluated here.^9^

MacKeith et al. found that, in general, the 0° endoscopes reached the highest temperatures in relation to the 30° and 70° endoscopes of the same caliber used, with the 70° endoscopes generating significantly more heat than the 30° endoscopes.^3^ However, the methods of measurements were different from our study, as we said before.

When evaluating the anatomical location where the light beams illuminated, we obtained greater temperature variations in the EAC of the right and left temporal bones compared to the round window in general. This difference can probably be attributed to the greater exposure of the EAC to the heat of the external environment heated by the thermal blanket used; in addition to that, the EAC is a compartment where the heat has more difficult to dissipate, while the RW, due to the communication of the tympanic cavity with the mastoid cells, may be less impacted, as heat dissipates in the mastoid cells. We must consider that in endoscopic surgeries for chronic otitis, the mastoid is usually less pneumatized and sometimes obliterated in relation to the tympanic cavity, increasing the chances of higher heat concentration in the middle and inner ear structures. This is a warning for otological surgeons not to touch the tip of rigid endoscopes to the EAC and not to spend too long with rigid endoscope inside the EAC.

We also believe that the greater distance between the endoscope tip and the thermometer probe, this distance being greater in the RW, may have influenced our results, as we introduced the endoscope into the EAC and the thermometer into the RW via posterior tympanotomy, but with the removal of the tympanic membrane, to maintain the visibility of the middle ear structures. In addition, the RW was not completely exposed to the light beam in many situations, mainly due to anatomical accidents involving the region.^3^

We noticed in our study that the temperature increased more sharply in the first minutes after turning on the light sources and, over time, this speed decreased, tending to reach temperature stability.

Accordingly, rapid temperature rises in the initial seconds of measurements are seen in the literature. Reaching 80% of the maximum temperature can happen on average 35 s after turning on the light source at the endoscope tip, as well as a rapid cooling of endoscopes when turning off the light source, with loss of 80% of the heat in 23 s, can occur.^3^

Other studies show similar results, but with more pronounced speed in the initial seconds, reaching high temperatures in the first 30–124 s, both with LED and xenon sources. Besides, they describe an important reduction in temperature after turning off the light source and applying local suction close to the endoscope tip. Thus, they suggest performing periodic removal of the endoscope to allow tissue cooling as an effective method, given the observation of a rapid decrease in temperature when the light source is turned off.^2^

Using temporal bones from cadavers in our study, we faced some limitations. These pieces do not allow reproduction of blood flow, which could dissipate more heat and cool the tissues, as blood perfusion limits the rise in temperature. Furthermore, we did not faithfully reproduce surgical techniques, but we evaluated the temperature rise statically, with the endoscopes kept in the same position during the 10 min of each measurement, which can also generate more heat compared to the surgical procedure itself, with its dynamics and recurrent removal of the endoscopes from the surgical field, allowing them to cool. And as a limitation of the literature, we identified that there are few studies that analyze the temperature in the manipulated tissue, with most registering temperatures at the endoscope tip itself.^2^, ^7^, ^8^

Even so, we believe that reproducing this study in temporal bones of cadavers allowed us to analyze the influence of different types of endoscopes and light sources in otologic surgeries, comparing the behavior of temperature elevations and their possible consequences in the tissues, so we can try to find solutions aimed at reducing the temperature and the risks to the tissues in the endoscopic surgery of the ear. Furthermore, we used the cadaver’s temporal bone in body temperature to simulate thermal variation and not just the initial and final temperature.

The literature suggests some measures necessary to limit the risks of tissue thermal injury, such as keeping the light source at the lowest effective intensity possible, not keeping the endoscope tip too close to the tissues or touching them, and constantly moving the endoscope to dissipate more heat.^7^, ^12^

Liu at al., analyzing the increase in the temperature of the round window in guinea pigs and the change in auditory evoked potential by exposure to a CO_2_ laser at an intensity of 10 W, 3-mm of distance at a cochleostomy, they found an increase of 8 °C, which was enough to induce hearing loss and in the histological study of these guinea pigs, they found damage to outer hair cells.^13^ This shows that little increase of temperature damages cochlear hair cells with hearing loss in animals. We consider that our work provides important information to raise awareness about the possibility of injury to the inner ear when using a 4-mm rigid endoscope in otological surgeries, especially for a longer period of time and with the tip of the endoscope close to the round window.

More studies are needed to deepen the research, especially in the living body to reproduce the thermal variation more faithfully and reduce these limitations.^1^ Therefore, we suggest a preference for small-caliber endoscopes, with LED light and at the lowest feasible intensity possible for performing a surgical procedure with lower risks.

## Conclusion

We concluded that endoscope and light source characteristics can influence the thermal variation and the risk of injury to the tissues manipulated during endoscopic ear surgery. Large-caliber endoscopes, associated with xenon and halogen light sources at maximum intensity, generate more heat.

## Funding

No funding was received for this research.

## Conflicts of interest

All authors have seen and approved this manuscript. The authors declare no conflicts of interest. The datasets generated and analyzed during the current study are available from the corresponding author on reasonable request.
